# Unimproved water sources and open defecation are associated with active trachoma in children in internally displaced persons camps in the Darfur States of Sudan

**DOI:** 10.1093/trstmh/trz042

**Published:** 2019-07-26

**Authors:** Colin K Macleod, Kamal Hashim Binnawi, Balgesa Elkheir Elshafie, Husam Eldin Sadig, Awad Hassan, Naomi Cocks, Rebecca Willis, Brian Chu, Anthony W Solomon, for the Global Trachoma Mapping Project

**Affiliations:** 1 Clinical Research Department, London School of Hygiene & Tropical Medicine, Keppel Street, London, UK; 2 National Program for Prevention of Blindness, Federal Ministry of Health, PO Box 303 Khartoum, Sudan; 3 Department of Ophthalmology, Al Neelain University, Khartoum, Sudan; 4 Faculty of Mathematical Sciences and Statistics, Al-Neelain University, Sudan; 5 Sightsavers, Nile Avenue, Khartoum, Sudan; 6 Task Force for Global Health, 330 W Ponce de Leon Ave, Decatur, GA, USA; 7 Department of Control of Neglected Tropical Diseases, World Health Organization, Geneva, Switzerland

**Keywords:** Darfur, Global Trachoma Mapping Project, prevalence, Sudan, trachoma, trichiasis

## Abstract

**Purpose:**

To estimate the proportion of children with trachomatous inflammation—follicular (TF) and adults with trachomatous trichiasis (TT) in internally displaced persons (IDP) camps in the Darfur States of Sudan and to evaluate associated risk factors.

**Methods:**

IDP camps were identified from government census data. We conducted a subanalysis of data collected in these camps during 2014–2015 as part of surveys covering 37 districts of the Darfur States within the Global Trachoma Mapping Project. A random-effects hierarchical model was used to evaluate factors associated with TF in children or TT in adults.

**Results:**

Thirty-six IDP camps were represented in the survey data, in which 1926 children aged 1–9 y were examined, of whom 38 (8%) had TF. Poor sanitation, younger age and living in a household that purchased water from a vendor were associated with TF in children aged 1–9 y. Of 2139 individuals examined aged ≥15 y, 16 (0.7%) had TT. TT was strongly independently associated with being older and living alone.

**Conclusion:**

Trachoma is found at low levels in these camps, but still at levels where intervention is needed. Disease elimination in conflict-related settings presents a unique challenge for the trachoma community, and may require an innovative approach. Understanding how best to undertake trachoma elimination interventions in these areas should be prioritized.

## Introduction

The United Nations Office for Coordination of Humanitarian Affairs (UNOCHA) estimates that in 2014 more than 59 million people worldwide were displaced from their homes due to conflict or insecurity.^1^ Over 38 million of these were internally displaced persons (IDPs) still living within the borders of their own country.^2^ Displaced persons present unique challenges to health systems, having an increased risk of infectious disease,^3–5^ malnutrition related to food and water insecurity,^6–8^ trauma-related psychiatric disorders^9–11^ and maternal mortality.^12,13^ Even where healthcare needs could otherwise be managed, large-scale interventions are made difficult by ongoing risks of violence^3,14,15^ and the itinerant nature of the population.

Trachoma is an eye disease that blinds through recurrent^16,17^ conjunctival infection with the bacterium *Chlamydia trachomatis.* It is the most common infectious cause of blindness worldwide,^18^ affecting the world’s poorest and most vulnerable populations.^19^ Infection is spread from eye to eye directly by touch, or indirectly through fomites or eye-seeking flies of the *Musca* genus. Female *Musca* spp. flies preferentially lay eggs on human feces left exposed on the soil.^20^ Trachoma is associated with low levels of access to water, sanitation and hygiene (WASH), being found in areas of extreme poverty across the globe.^21^

WHO has targeted trachoma for elimination by 2020^22^ using the SAFE strategy. This consists of Surgery for those with advanced disease, Antibiotic treatment, Facial cleanliness and Environmental improvement in endemic areas.^23^ Recommendations for the A, F and E interventions are stratified by the prevalence of the sign trachomatous inflammation—follicular (TF), with (for example) areas having a prevalence of TF ≥10% in children aged 1–9 y requiring mass drug administration (MDA) of antibiotics, plus implementation of the F and E components of SAFE for at least 3 y before review.^21,24^ In theory, F reduces the community-level volume of infected secretions available for transfer from eye to eye^25^; E, which involves increasing access to water and sanitation, facilitates facial cleanliness and reduces the number of breeding sites for *Musca* spp.^26^ However, the evidence base for the F and E interventions is significantly weaker than that for S and A.^27–29^

The Darfur States in Sudan have been affected by conflict and population displacement since the outbreak of civil war in 2003. UNOCHA estimates that as of December 2015 there were 2.7 million displaced people in Darfur out of a total population of 8.8 million.^30,31^ Darfur comprises five states in the West of Sudan: North, East, West, South and Central Darfur.

Recent trachoma surveys conducted in the Darfur States justify antibiotic MDA in five districts found to have a high prevalence of TF in children. These surveys included IDP camps, and found that living in an IDP camp was a strong independent risk factor for TF, with children living in these camps having odds of TF 2.6 times higher than non-IDP camp-resident children.^32^ In this paper, we conduct a subanalysis of this IDP camp population to identify independent risk factors that might explain this increased risk of trachoma in the IDP camp population. We report the trachoma prevalence and between-camp risk factors associated with disease.

## Methods

### Study design

We conducted a secondary analysis of data from 27 cross-sectional population-based trachoma prevalence surveys carried out in the Darfur States during 2014–2015. Full details of the methodologies used in the original surveys are presented elswhere.^32–34^ IDP camps were identified from these primary data sets by cross-referencing government census data.

### Water and sanitation access

Household-level data on access to water supply for drinking and washing, and sanitation facilities, were collected by field teams at the time of the survey by a focused interview with the head of the household and by direct observation. Water and sanitation access was recorded by a combination of direct observation of structures and responses to questions, depending on whether local or remote structures were used; reported use was obtained by interviewing the head of the household. Recorders were trained to identify water and sanitation infrastructure and access categories based on the WHO/UNICEF Joint Monitoring Programme for Water Supply, Sanitation and Hygiene definitions, which were used up to 2015.^35,36^ GPS coordinates were recorded at the front entrance to each household.

### Climate variables

Data on local climate (at 2.5 arc-min resolution ~5 km) were downloaded from WorldClim BioClim variables (worldclim.org). Annual mean rainfall and mean temperature, maximum temperature of the hottest month, and major landcover type, were considered as variables that could potentially influence infection transmission and therefore disease prevalence. Values were extracted from available rasters at the cluster level, defined as the mean easting and mean northing GPS coordinates of all households in a given cluster.

### Statistical analysis

Multilevel logistic regression was used in order to account for a change in the variance in the outcome variable between the different population levels.^37^ Data were collected from units at three different levels (state, camp and household). Outcomes were evaluated for potential clustering of both TF and trachomatous trichiasis (TT) at state, camp and household levels. If no clustering was identified, a standard logistic regression model was used. Univariable associations were considered for inclusion in the multivariate model if p≤0.05 (Wald’s χ^2^ test). A stepwise-inclusion approach was used, with variables retained in the model if significant at the p≤0.05 level (likelihood ratio test). All risk factor analysis was carried out in Stata 10.2 (Stata Corp LP, College Station, TX, USA).

## Results

**Figure 1.**
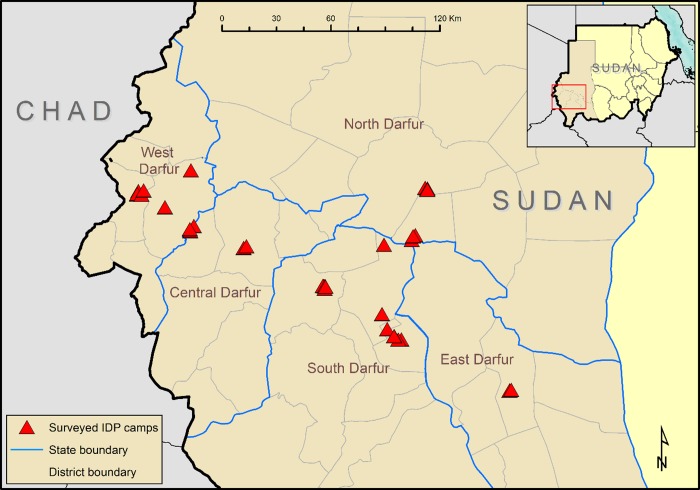
Internally displaced persons (IDP) camps surveyed as part of the Global Trachoma Mapping Project, Darfur States, Sudan, 2014–2015.

A total of 36 IDP camp clusters within 11 districts were identified in 27 surveys in the Darfur States (Table 1, Figure 1). In principle, an IDP camp should be a transient place of residence for individuals who have been forced to migrate due to (for example) civil unrest or famine. In reality, some camps here were settled decades ago, with a proportion inhabited by those who migrated from what is now South Sudan, and they may have little hope of return. Because of the method of selection we used, all included data were by definition from IDP camps that were formally recognized by the government of Sudan, and so were not likely to be recent settlements.

**Table 1.** Numbers sampled, examined, absent, refused and showing trachomatous inflammation—follicular (TF) or trachomatous trichiasis (TT), internally displaced persons camps, Darfur States, Sudan, 2014–2015

State	District (camp#)	Households	Examined	Absent	Refused/other	Total	1–9-y-olds			10–14-y-olds			≥15-y-olds		
							TF	TT	Examined	TF	TT	Examined	TF	TT	Examined
Central Darfur	Azoom	30	103	3	0	106	0	0	45	0	0	6	0	4	55
	Zalinji (1)	30	115	38	1	154	0	0	48	0	0	20	0	0	86
	Zalinji (2)	30	93	26	0	119	11	0	49	0	0	19	0	0	51
East Darfur	El Daein (East) (1)	30	130	0	0	130	4	0	76	0	0	13	0	0	41
	El Daein (East) (2)	30	94	19	2	115	0	0	47	0	0	15	0	0	53
	El Daein (East) (3)	29	97	56	0	153	0	0	63	0	0	21	0	0	69
North Darfur	El Fashir (1)	30	102	14	0	116	7	0	62	1	0	21	1	1	54
	El Fashir (2)	31	109	0	0	109	2	0	54	0	0	1	0	0	61
	El Fashir (3)	31	112	2	0	114	0	0	43	0	0	12	0	0	55
	El Fashir (4)	29	98	9	0	107	2	0	47	0	0	15	0	0	54
	Dar El Salam (1)	30	109	28	0	137	4	1	39	0	0	26	0	1	44
	Dar El Salam (2)	30	104	12	0	116	14	0	57	0	0	11	0	1	46
	Dar El Salam (3)	30	101	9	0	110	9	0	48	0	0	2	0	1	57
South Darfur	Kas (1)	30	114	22	0	136	1	0	47	0	0	9	0	0	53
	Kas (2)	31	101	29	0	130	7	0	43	1	0	15	0	0	80
	Kas (3)	31	140	0	0	140	6	0	24	1	0	12	0	0	66
	Kas (4)	30	144	4	0	148	1	0	56	0	0	9	0	0	52
	Kas (5)	30	147	15	0	162	13	0	72	0	0	8	0	1	56
	Kas (6)	30	135	16	0	151	6	0	50	0	0	13	0	1	67
	Nyala City (1)	30	116	33	0	149	6	0	69	0	0	8	0	1	63
	Belale (1)	30	109	0	0	109	6	0	67	0	0	11	0	2	70
	Belale (2)	31	113	25	0	138	0	0	69	1	0	29	0	0	64
	Belale (3)	29	82	20	0	102	1	0	74	0	0	11	0	0	66
	Belale (4)	30	117	0	0	117	1	0	69	0	0	16	0	1	64
	Unitty (1)	30	122	8	1^a^	131	0	0	47	0	0	22	0	0	62
	Unitty (2)	30	98	19	0	117	0	0	43	0	0	15	0	0	59
West Darfur	El Jinaina (1)	30	72	40	0	112	1	0	36	0	0	6	0	0	70
	El Jinaina (2)	30	139	26	0	165	1	0	74	0	0	19	0	0	72
	El Jinaina (3)	30	77	21	0	98	8	0	41	0	0	9	0	0	48
	El Jinaina (4)	30	96	40	0	136	21	0	59	0	0	14	0	0	63
	El Jinaina (5)	30	75	38	0	113	3	0	37	0	0	13	0	1	63
	El Jinaina (6)	29	85	23	0	108	3	0	41	0	0	17	0	0	50
	Kreanik (1)	29	133	8	0	141	0	0	71	0	0	16	0	0	54
	Kreanik (2)	30	103	36	0	139	0	0	65	0	0	13	0	0	61
	Kreanik (3)	30	96	29	0	125	0	0	49	0	0	20	0	1	56
	Kreanik (4)	30	96	7	0	103	0	0	45	0	0	4	0	0	54
**Total**		**1080**	**3877**	**675**	**4**	**4556**	**138**	**1**	**1823**	**4**	**0**	**362**	**1**	**16**	**1692**

^a^Other: reason not specified

A total of 1080 households and 4556 individuals were enumerated in the 36 IDP camp clusters (Table 2), of whom 3877 individuals (85%) were present and consented to examination. A total of 1926 children aged 1–9 y were enumerated over all identified IDP camps, with 1823 (95%) present and consenting to examination. The median age of participants of all ages was 13 y (range 1–100 y) and 56% were female.

**Table 2.** Study population characteristics

Parameter	Number, N
States	5
Districts	13
Internally displaced persons camps	36
Households	1080
Population sampled	4556
Female (%)	2548 (56%)
Children aged 1–9 y	1926
Median individuals examined/camp (IQR)	111.5 (98–122)
Median children aged 1–9 y/camp (IQR)	42.5 (23–47.5)
Camp land cover grassland (%)	28 (78%)
Median annual rainfall (mm)	453
Median annual mean temperature (°C)	25.5
Median temperature of hottest month (°C)	38.6
Median individuals/household	3
Median number of 1–9-y-olds/household	2

### Proportion of TF

Of 1823 children aged 1–9 y examined, 138 (8%) had TF and 4 (0.2%) had trachomatous inflammation—intense (TI). All four cases of TI also had TF. The proportion of children with TF aged 1–9 y in each camp ranged from 0 to 40%. No cases of TF were found in 11 of the 36 camps.

### Clustering of TF

A null model for TF adjusted for age and gender showed statistically significant clustering at state, camp and household levels. The adjusted standard deviation in the odds due to between-state clustering was 0.38 (SE 0.20, p=0.01), due to between-camp clustering was 1.54 (SE 0.28, p<0.0001), and due to between-household clustering was 2.35 (SE 0.31, p<0.0001). The model adjusting for clustering at household level only was a better fit than models also accounting for state and camp clustering. All subsequent analyses on TF presented in this paper are from two-level hierarchical models with adjustment for household-level clustering.

### Proportion of TT

Of 2139 individuals examined aged ≥15 y, 16 (0.7%) had TT. The camp-level prevalence of TT ranged from 0% up to a maximum of 7.3% (4/55) of those aged ≥15 y in an IDP camp in the Azoom district of Central Darfur.

### Clustering of TT

A null model for TT adjusted for age and gender showed no statistically significant clustering at state, camp or household levels. All subsequent analyses on TT presented in this paper are from logistic regression models.

### Factors associated with TF in children aged 1–9 y

Univariable associations of TF are presented in Table 3. In the final multivariable model, TF was independently associated with being aged 1–4 y (OR 1.7, 95% CI 1.1 to 2.7); practicing open defecation (OR 3.1, 95% CI 1.1 to 8.6) and accessing a shared (as opposed to a private household) latrine (OR 3.0, 95% CI 1.1 to 8.5). There was also a strong independent association between TF and obtaining household drinking water from a water vendor (OR 9.9, 95% CI 2.1 to 46.8) compared with obtaining water from an improved source (Table 4).

**Table 3.** Univariable associations of trachomatous inflammation–follicular (TF) in children aged 1–9 y, internally displaced persons camps, Darfur States, Sudan, 2014–2015

Individual	TF (%)	N (%)	OR (95% CI)^a^	p-value^b^
Age				
1–4 y	81 (8.9)	912 (50.0)	1.71 (1.07–2.72)	**<0.0001**
5–9 y	57 (6.3)	911 (50.0)		
Gender				
Male	68 (7.4)	923 (50.6)	1	0.754
Female	70 (7.8)	900 (49.4)	1.07 (0.69–1.68)	
**Household**				
Household size				
≥8 members	19 (8.0)	236 (13.0)	1.28 (0.49–3.33)	0.613
1–7 members	119 (7.5)	1587 (87.0)	1	
Number of resident children aged 1–9 y				
≥5	12 (6.0)	200 (11.0)	0.61 (0.19–1.91)	0.395
1–4	126 (7.8)	1623 (89.0)	1	
*Water access* ^c^				
Main source of water for drinking				
Improved source^d^	69 (5.7)	1210 (66.4)	1	**0.0095**
Unimproved source^e^	12 (3.5)	343 (18.8)	0.4 (0.1–2.3)	
Water vendor	57 (21.1)	270 (14.8)	7.8 (1.7–34.9)	
Time to main source of drinking water				
Up to 30 min round-trip	103 (8.0)	3245 (71.2)	1	0.2088
≥30 min	35 (6.5)	1311 (28.8)	0.4 (0.1–1.8)	
Main source of water for washing				
Improved source^d^	69 (5.7)	1209 (66.3)	1	0.0094
Unimproved source^e^	12 (3.5)	344 (18.9)	0.4 (0.1–2.2)	
Water vendor	57 (21.1)	270 (14.8)	7.8 (1.7–34.8)	
Time to main source of washing water				
Up to 30 min round-trip	103 (8.0)	1284 (70.4)	1	0.2514
≥30 minutes	35 (6.5)	539 (29.6)	0.4 (0.1–2.0)	
*Latrine access* ^c^				
Private latrine	38 (5.2)	744 (40.8)	1	**0.0357**
Latrine facilities absent (open defecation)	43 (6.4)	674 (37.0)	2.86 (1.02–8.05)	
Shared latrine access	57 (14.1)	405 (22.2)	3.27 (1.15–9.25)	
Latrine type^f^				
Pit latrine with slab (improved pit latrine)	20 (4.7)	425 (23.3)	1	0.1691
Pit latrine without slab (unimproved pit latrine)	73 (10.0)	728 (39.9)	1.3 (0.4–4.0)	
No facilities, bush or field	45 (6.8)	660 (36.2)	2.9 (0.9–9.5)	
Other^g^	0 (0.0)	10 (0.5)	-	
**Camp**				
*Climate/environment* ^k^				
Rainfall^l^				
Desert (annual rainfall <500 mm)	95 (8.2)	1153 (63.3)	1.5 (0.7–3.2)	0.2445
≥500 mm	43 (6.4)	670 (36.8)	1	
Mean temperature annually^l^				
≥25°C	95 (7.6)	1256 (68.9)	1.1 (0.2–5.9)	0.9067
<25°C	43 (7.6)	567 (31.1)	1	
Maximum temperature of hottest month^l^				
≥40°C	15 (8.9)	169 (9.3)	1.3 (0.4–3.9)	0.6945
<40°C	123 (7.4)	1654 (90.7)	1	
*Major land cover type*				
Open shrubland	34 (14.1)	241 (13.2)	4.1 (0.6–29.5)	0.4371
Grassland	100 (6.9)	1444 (79.2)	1	
Croplands/natural vegetation mosaic	2 (2.1)	94 (5.2)	0.3 (0.1–8.9)	
Barren or sparsely vegetated	2 (4.6)	44 (2.4)	1.3 (0.1–87.8)	

^a^Unadjusted OR and 95% CI from two-level mixed effects logistic regression

^b^p-value from Wald’s χ^2^, statistically significant associations highlighted in bold (p≤0.05)

^c^from focused interview with head of household

^d^Improved water source—piped water into dwelling, piped water to yard/plot, public tap or standpipe, tubewell or borehole, protected dug well, protected spring, rainwater; 15 households had different washing/drinking water sources

^e^Unprotected spring, unprotected dug well, surface water

^f^direct observation by researchers

^g^Latrine subtype not specified further

^k^extracted from 2.5 arc-min (~5 km at the equator) raster data from worldclim.org

^i^No association in linear model, categorical data presented here

^h^Open shrublands, cropland/natural vegetation mosaic, barren or sparsely vegetated

**Table 4.** Multivariable logistic regression model of trachomatous inflammation—follicular (TF) in children aged 1–9 y, internally displaced persons camps, Darfur States, Sudan, 2014–2015

Variable	OR (95% CI)^a^	p-value^b^
**Individual**		
Age 1–4 y (compared with 5–9 y)	1.7 (1.0–2.7)	0.027
**Household**		
Main source of water for drinking^c^		
Improved source^d^	1	0.0136
Unimproved source^e^	0.4 (0.2–1.2)	
Water vendor	8.5 (4.1–21.0)	
*Latrine access* ^d^		
Private latrine	1	0.024
Latrine facilities absent (use of open defecation)	3.1 (1.1–8.6)	
Shared latrine access	3.0 (1.1–8.5)	

^a^Adjusted OR and 95% CI from two-level mixed effects logistic regression; sex included in model a priori

^b^p-value from likelihood ratio test for variable nested in full model

^c^from focused interview with head of household

^d^Piped water into dwelling, piped water to yard/plot, public tap or standpipe, tubewell or borehole, protected dug well, protected spring, rainwater; 15 households had different washing/drinking water sources.

^e^Unprotected spring, unprotected dug well, surface water

In IDP camps in Sudan it is common for households to rely on delivery of water in recycled oil barrels. Households obtaining water from vendors were present in 6 of the 36 camps surveyed, with 96% (173/181) of households surveyed in these 6 camps identifying such water vendors as their water source. In these 6 camps the proportion of children aged 1–9 y with TF was 21.1% (57/270) compared with 5.2% (81/1472) in the other 30 camps, the difference being highly statistically significant (p<0.0001, χ^2^ test).

### Factors associated with TT in those aged ≥15 y

Univariable associations with TT are presented in Table 5. In the final multivariable model, TT was strongly independently associated with being aged ≥40 y (OR 22.0, 95% CI 2.8 to 171.6) and living alone (OR 6.9, 95% CI 2.1 to 22.7, Table 6).

**Table 5.** Univariable associations of trachomatous trichiasis (TT) in those aged ≥15 y, internally displaced persons camps, Darfur States, Sudan, 2014–2015

Individual	TT (%)	N (%)	OR (95% CI)^a^	p-value^b^
Age				
≥40 y	15 (2.6)	575 (34.0)	31.9 (4.2–244.3)	**0.0009**
15–39 y	1 (0.1)	1117 (66.0)	1	
Gender				
Female	13 (1.1)	1183 (69.9)	1.9 (0.5–6.6)	0.3284
Male	3 (0.6)	509 (30.1)	1	
**Household**				
Household members				
At least one other person	10 (0.6)	1609 (95.0)	1	
Lives alone	6 (7.2)	83 (4.9)	14.6 (4.8–44.5)	**<0.0001**
Number of children aged 1–9 y in the household				
≥1	5 (0.4)	1236 (73.0)	1	**0.0007**
0	11 (2.4)	456 (27.0)	6.5 (2.2–19.2)	
*Water access* ^c^				
Main source of water for drinking				
Improved source^d^	13 (1.1)	1131 (66.8)	1	0.4893
Unimproved source^e^	1 (0.3)	313 (18.5)	0.3 (0.0–2.4)	
Water vendor	2 (0.8)	248 (14.7)	0.7 (0.1–3.7)	
Time to main source of drinking water				
Up to 30 min round-trip	8 (0.7)	1241 (73.4)	1	
≥30 min	8 (1.8)	451 (26.7)	2.7 (0.9–7.8)	**0.0683**
Main source of water for washing				
Improved source^d^	13 (1.1)	1131 (66.8)	1	0.4894
Unimproved source^e^	1 (0.3)	313 (18.5)	0.3 (0.0–2.4)	
Water vendor	2 (0.8)	248 (14.7)	0.7 (0.1–3.7)	
Time to main source of washing water				
Less than 30 min round-trip	8 (0.6)	1247 (73.7)	1	**0.062**
≥30 min	8 (1.8)	445 (26.3)	2.8 (0.9–8.0)	
*Latrine access* ^c^				
Private latrine	6 (0.8)	723 (42.7)	1	0.6169
Shared latrine access	5 (1.3)	388 (22.9)	1.7 (0.5–6.5)	
Latrine facilities absent (use of open defecation)	5 (0.9)	581 (34.3)	0.8 (0.2–3.4)	
Latrine type^f^				
Pit latrine with slab (improved pit latrine)	3 (0.7)	417 (24.7)	1	0.737
Pit latrine without slab (unimproved pit latrine)	8 (1.1)	706 (41.7)	1.5 (0.4–6.4)	
No facilities, bush or field	5 (0.9)	569 (33.6)	0.9 (0.2–5.0)	
Other^g^	0 (0.0)	9 (0.5)	-	
Camp				
*Climate/environment* ^k^				
Rainfall^l^				
Desert (annual rainfall <500 mm)	6 (0.6)	1095 (64.7)	0.3 (0.1–0.9)	
≥500 mm	10 (1.7)	597 (35.3)	1	**0.0296**
Mean temperature annually^l^				
≥25°C	11 (0.9)	1168 (69.0)	0.9	
<25°C	5 (0.9)	524 (31.0)	1	0.9443
Maximum temperature of hottest month^l^				
≥38°C	11 (1.0)	574 (33.9)	1.1 (0.3–3.8)	0.8746
<38°C	5 (0.9)	1118 (66.1)	1	
*Major land cover type*				
Grasslands	12 (0.9)	1311 (77.5)	1	0.7379
Other^g^	4 (1.0)	381 (22.5)	1.3 (0.3–4.8)	

^a^Unadjusted OR and 95% CI from logistic regression

^b^p-value from Wald’s χ^2^, statistically significant associations highlighted in bold (p≤0.05)

^c^Focused interview with head of household

^d^Improved water source—piped water into dwelling, piped water to yard/plot, public tap or standpipe, Tubewell or borehole, protected dug well, protected spring, rainwater; 15 households had different washing/drinking water sources

^e^Unprotected spring, unprotected dug well, surface water

^f^Direct observation by data recorders

^g^Not specified; no TT outcome in group, so excluded from univariable analysis.

^k^Extracted from 2.5 arc-min (~5 km at equator) raster data from worldclim.org

^i^No association in linear model, categorical data presented here

^g^Open shrublands, cropland/natural vegetation mosaic, barren or sparsely vegetated

**Table 6.** Multivariable logistic regression model of trachomatous trichiasis (TT) in those aged ≥15 y, internally displaced persons camps, Darfur States, Sudan, 2014–2015

Variable	OR (95% CI)^a^	p-value^b^
Age ≥40 y	25.4 (3.2–200.0)	0.002
Living alone	6.9 (2.1–22.7)	0.001

^a^Adjusted OR and 95% CI from two-level mixed effects logistic regression; sex included a priori

^b^p-value from likelihood ratio test for variable nested in full model

## Discussion

We found clustering of TF at state, IDP camp and household levels. The strongest effect of clustering was found at household level, consistent with the evidence presented by other studies,^38–42^ and a two-level hierarchical model was used to account for this. At an individual level, in IDP camps in Sudan, younger age was strongly associated with TF, another common finding in the trachoma literature from other contexts.^38,39,42,43^ We found no association between TF and gender, in keeping with trachoma’s close-contact mode of transmission, in which exposure would be expected to be similar in children of both genders;^44–46^ however, we included gender in all models a priori.

Household access to a private latrine of any kind was associated with decreased odds of TF. This could be considered a small validation of the WHO/UNICEF Joint Monitoring Programme for Water Supply, Sanitation and Hygiene’s definition of an improved latrine as one used by a single household (among several other criteria). Private latrine ownership is believed to confer a greater incentive to keep the latrine clean and well maintained, which in turn encourages more consistent use. However, the literature to support this having a protective association against trachoma is lacking: a number of studies suggest that the use of any latrine is protective, without identifying any additional benefit conferred by the latrine being private.^47–49^ In addition, several studies have suggested that community WASH coverage thresholds, rather than household-level WASH outcomes, are a better indicator of the protective association of access to sanitation, probably because of the decreased availability of feces in open areas to facilitate the spread of *Musca* spp. flies.^50,51^ In our data, similar odds of TF were observed between residents of households that practiced open defecation and residents of households using shared latrines. It is of course possible that the protective association of private latrine access here is mediated through other (unmeasured) factors, such as the health or educational advantages enjoyed by those with sufficient resources to enable maintenance of a private latrine.

At household level, sourcing drinking water primarily from a water vendor was strongly independently associated with TF in children. This relationship was also seen using the main source of washing water as the explanatory variable, although both source of drinking water and source of washing water were not included in the full model due to collinearity between the two variables (as most households’ sources of drinking and washing water were the same). A higher number of people living in the household was not associated with TF. Anecdotally, oil barrels for holding water are stored in the home and water from them is used for all drinking, cooking and cleaning by the household; in this context it would be understandable if use of the water for personal hygiene purposes was not prioritized because of the associated cost. However, the relationship between water cost or distance-to-source and use is known to be complex, and so we are wary of overinterpreting these data.^52,53^

IDP camps present a difficulty for trachoma elimination ambitions. There is relatively little experience in the implementation of the SAFE strategy in conflict-related settings such as these, and the question of whether decisions regarding interventions in these camps should be considered under the same guidance as non-IDP populations arises. Despite the IDP camps being seen as relative hotspots for trachoma, existing WHO guidelines make no recommendations as to how healthcare providers should respond. Understanding how best to undertake trachoma elimination interventions in these areas has to be a priority on disease control (as well as humanitarian) grounds.

Studies on the efficacy of health outcomes of hygiene and sanitation interventions in humanitarian crises are lacking, with published studies usually presenting data on the incidence of diarrheal illness. Few studies look at specific WASH interventions, including the fidelity of implementation and levels of utilization, so that to date, evidence of the impact of such interventions in this setting is scarce.^54–61^

In the case of trachoma, the association between disease and limited water access has been described before,^45,62,63^ but with notable exceptions where such a relationship was not evident;^64–67^ the disparity probably relates to the fact that ready access to water does not necessarily mean frequent use of water for personal hygiene.^68^ Beyond associated data, studies that demonstrate a significant impact of water or sanitation interventions on trachoma prevalence are still needed. Implementing sanitation interventions, like implementing the other components of the SAFE strategy, is likely to present specific challenges in the IDP camp setting, although this should not discourage us from working towards achievement of the Sustainable Development Goals for equitable and sustainable access to water and sanitation.

## Appendix

The Global Trachoma Mapping Project Investigators are: Agatha Aboe (1,11), Liknaw Adamu (4), Wondu Alemayehu (4,5), Menbere Alemu (4), Neal D. E. Alexander (9), Ana Bakhtiari (2,9), Berhanu Bero (4), Sarah Bovill (8), Simon J. Brooker (1,6), Simon Bush (7,8), Brian K. Chu (2,9), Paul Courtright (1,3,4,7,11), Michael Dejene (3), Paul M. Emerson (1,6,7), Rebecca M. Flueckiger (2), Allen Foster (1,7), Solomon Gadisa (4), Katherine Gass (6,9), Teshome Gebre (4), Zelalem Habtamu (4), Danny Haddad (1,6,7,8), Erik Harvey (1,6,10), Dominic Haslam (8), Khumbo Kalua (5), Amir B. Kello (4,5), Jonathan D. King (6,10,11), Richard Le Mesurier (4,7), Susan Lewallen (4,11), Thomas M. Lietman (10), Chad MacArthur (6,11), Colin Macleod (3,9), Silvio P. Mariotti (7,11), Anna Massey (8), Els Mathieu (6,11), Siobhain McCullagh (8), Addis Mekasha (4), Tom Millar (4,8), Caleb Mpyet (3,5), Beatriz Muñoz (6,9), Jeremiah Ngondi (1,3,6,11), Stephanie Ogden (6), Alex Pavluck (2,4,10), Joseph Pearce (10), Serge Resnikoff (1), Virginia Sarah (4), Boubacar Sarr (5), Alemayehu Sisay (4), Jennifer L. Smith (11), Anthony W. Solomon (1,2,3,4,5,6,7,8,9,10,11), Jo Thomson (4); Sheila K. West (1,10,11), Rebecca Willis (2,9).

1. Advisory Committee; 2. Information Technology, Geographical Information Systems and Data Processing; 3. Epidemiological Support; 4. Ethiopia Pilot Team; 5. Master Grader Trainers; 6. Methodologies Working Group; 7. Prioritisation Working Group; 8. Proposal Development, Finances and Logistics; 9. Statistics and Data Analysis; 10. Tools Working Group; 11. Training Working Group.
